# 

**Published:** 1882-07

**Authors:** 


### *Pennsylvania State Dental Society.*

The Fourteenth Annual Session of this society will be held July 25th, 26th, and 27th, at Williamsport, Pa. Arrangements have been made with the rail roads by which tickets can be secured at reduced rates, by presenting orders obtained from the Corresponding Secretary. The Park Hotel has reduced its figures from three to two dollars per day, to guest attending Convention.

Williamsport is delightfully situated on the North branch of the Susquehanna river, 70 miles North-west of Harrisburg, and is 900 feet above the sea; surrounded by some of Pennsylvania's finest mountain scenery, making it exceedingly picturesque, and with health giving atmosphere makes it an exceedingly pleasant resort.

The following programme has been prepared by the executive committee:

*Essays.*—President's Address, C. B. Ansart, D. D. S., Oil City. "Lesions of the Alveolus," N. E. Magill, Erie. "The Influence of Constitutional Defects upon Dental Tissues," by C. S. Beck, M. D., D. D.S., Wilksbare. "Nervous Force and Nervous Lesions" illustrated by vivisections, by N. C. Barrett, M. D., D. D. S., Buffalo. "What Constitutes Success in Dental Practice," by D. T. Nay, D. D. S., Bedford. "Reaction of Dentine with Special Relation to the Combination of Gold and Amalgam in Approximate Cavities," by H. C. Register, M. D. Philadelphia "Adjusting Artificial Crowns to Roots of Natural Teeth," by W. H. Fundenberg, D, D. S., Pittsburg.

*Demonstrations.*—"Pivoting all Porcelain Crowns," by N. G. A. Bonwill, M. D., Philadelphia. "Pivoting Weston's Crowns." "The Application of Gold and Amalgam in Combination," by H. C. Register, M. D., Philadelphia.

Baltimoreans can secure excursion tickets by presenting orders at the following Stations, Union, Calvert, and Corner Baltimore and Calvert Sts.

W. H. Fundenberg,

Pittsburg, Pa. *Corresponding Sec'y.*

---

*National Dental Association of the United States of America.*

The next meeting of the National Dental Association of the United States of America will be held in Washington, D. C., in the lecture hall of the National Museum of the Smithsonian Institution on the 3rd, 4th and 5th of August, 1882.

The meetings of the Association in Washington are quadrennial and international. A cordial invitation is extended to all members of the Profession in this and other countries to be present at this meeting.

R. Finley Hunt. D. D. S., *Sec'y.*

---

*American Dental Association.*

The Twenty-second Annual Session of the American Dental Association will be held at Cincinnati, commencing Tuesdsy August 1st, 1882.

Geo. H. Cushing, *Rec. Sec.*

### *Southern Dental Association.*

The Fourteenth Annual Meeting of the Southern Dental Association will be held in the building of the Baltimore College of Dental Surgery, Corner of Eutaw and Franklin Streets, Baltimore, Md., commencing Tuesday, August 8th prox. A cordial welcome is extended to all dentists to attend.

E. S. Chisholm, *Pres.*
W. H. Hoffman, *Sec'y.*

---

### *New York State Dental Society.*

The Fourteenth Annual Meeting of the above society, was held at Albany, N. Y., May 10th and 11th, 1882.

The following officers were elected for the ensuing year:

President, L. S. Straw, Newberg; Vice-president, Frank French, Rochester; Treasurer, A. H. Brockaway, Brooklyn; Secretary, J. Edw. Line, Rochester; Correspondent, W. A. Atkinson, New York.

J. Edw. Line, *Secretary.*

---

### EDITORIAL, ETC.

### To Readers and Correspondents.

Communications intended for publication, and exchange journals and papers, should be addressed to the EDITOR OF THE AMERICAN JOURNAL OF DENTAL SCIENCE, 259 N. Eutaw St., Baltimore, Md. All letters relating to the business of the journal. or containing cash remittances, to be directed to Messrs. SNOWDEN & COWMAN. Articles for publication should be received by the editor at least three weeks previous to the first of the month on which the number in which it is designed for them to appear, is issued.

### EDITORIAL, ETC.

The Connection of Dental with Medical Education .... 135
Maryland University Dental Department Building .... 139
The Amalgam Question .... 140
The Two Editors .... 142

### MONTHLY SUMMARY.

The Medicinal Value of Vegetables .... 142
Is this a New Dental Disease? .... 143
Removal of Foreign Bodies from the Ear .... 143
Congenital Teeth .... 144

☞ The American Journal of Dental Science will contain fifty pages of reading matter in each number, making a volume of about *Six Hundred* pages,

EXCLUSIVE OF ADVERTISEMENTS.

THE

### AMERICAN JOURNAL

OF

### DENTAL SCIENCE.

---

The *Journal* is issued on the first of each month and contains not less than *fifty* pages of reading matter in each number, exclusive of advertisements, making a volume of *six hundred* pages per annum.

In each number will be found Original Communications, Correspondence, Selected Articles, Monthly Summary, Bibliographical Notices and Editorial Matter, and every effort will be exerted to render the *Journal* worthy of the patronage of the Dental profession.

A generous co-operation is respectfully solicited from the members of the profession; and as the *Journal* has now a printing office of its own, which materially lessens the cost of its publication, it will be issued at the reduced subscription price of

**$2.50 Per Annum in Advance.**

Communications are respectfully solicited on all subjects pertaining to the practice of dentistry, as well as reports of cases occurring in dental practice.

---

### RATES OF ADVERTISING.

| | 1 MO. | 2 MO. | 3 MO. | 6 MO. | 12 MO. |
|---|---|---|---|---|---|
| 1 PAGE. | $15 00 | $22 50 | $30 00 | $50 00 | $100 00 |
| ½ " | 10 00 | 15 00 | 20 00 | 30 00 | 50 00 |
| ¼ " | 7 00 | 11 00 | 15 00 | 20 00 | 30 00 |
| ⅛ " | 5 00 | 8 00 | 11 00 | 15 00 | 20 00 |

All original communications designed for publication, works for review, new instruments for notice, exchanges, &c., should be directed to the editor, 259 N Eutaw St., Baltimore, Md.

All advertisements and subscriptions should be addressed to

SNOWDEN & COWMAN,

*Publishers of the Am. Journal of Dental Science.*

86 W. FAYETTE ST. BET. CHARLES & LIBERTY STS.,

BALTIMORE, MD.

